# Resolving deep relationships of PACMAD grasses: a phylogenomic approach

**DOI:** 10.1186/s12870-015-0563-9

**Published:** 2015-07-11

**Authors:** Joseph L. Cotton, William P. Wysocki, Lynn G. Clark, Scot A. Kelchner, J Chris Pires, Patrick P. Edger, Dustin Mayfield-Jones, Melvin R. Duvall

**Affiliations:** Biological Sciences, Northern Illinois University, 1425 W. Lincoln Hwy, DeKalb, Illinois 60115-2861 U.S.A; Ecology, Evolution and Organismal Biology, Iowa State University, 251 Bessey Hall, Ames, Iowa 50011-1020 U.S.A; Division of Biological Sciences, Bond Life Sciences Center, University of Missouri, Columbia, MO 65211-7310 U.S.A; Department of Plant and Microbial Biology, University of California, Berkeley, California, 94720 U.S.A; Biological Sciences, Idaho State University, 921 S. 8th Ave, Pocatello, Idaho 83209 U.S.A; Donald Danforth Plant Science Center, 975 North Warson Rd, St. Louis, Missouri 63132 U.S.A

**Keywords:** Complete plastome, Divergence estimates, PACMAD Clade, Panicoideae, Phylogenomics, Rapid radiation

## Abstract

**Background:**

Plastome sequences for 18 species of the PACMAD grasses (subfamilies Panicoideae, Aristidoideae, Chloridoideae, Micrairoideae, Arundinoideae, Danthonioideae) were analyzed phylogenomically. Next generation sequencing methods were used to provide complete plastome sequences for 12 species. Sanger sequencing was performed to determine the plastome of one species, *Hakonechloa macra*, to provide a reference for annotation. These analyses were conducted to resolve deep subfamilial relationships within the clade. Divergence estimates were assessed to determine potential factors that led to the rapid radiation of this lineage and its dominance of warmer open habitats.

**Results:**

New plastomes were completely sequenced and characterized for 13 PACMAD species. An autapomorphic ~1140 bp deletion was found in *Hakonechloa macra* putatively pseudogenizing *rpl14* and eliminating *rpl16* from this plastome. Phylogenomic analyses support Panicoideae as the sister group to the ACMAD clade. Complete plastome sequences provide greater support at deep nodes within the PACMAD clade. The initial diversification of PACMAD subfamilies was estimated to occur at 32.4 mya.

**Conclusions:**

Phylogenomic analyses of complete plastomes provides resolution for deep relationships of PACMAD grasses. The divergence estimate of 32.4 mya at the crown node of the PACMAD clade coincides with the Eocene-Oligocene Transition (EOT). The Eocene was a period of global cooling and drying, which led to forest fragmentation and the expansion of open habitats now dominated by these grasses. Understanding how these grasses are related and determining a cause for their rapid radiation allows for future predictions of grassland distribution in the face of a changing global climate.

**Electronic supplementary material:**

The online version of this article (doi:10.1186/s12870-015-0563-9) contains supplementary material, which is available to authorized users.

## Background

Poaceae have been the subject of numerous phylogenetic studies due to their economic and ecological importance, as well as their dominance in major terrestrial biomes [1–5]. The current phylogenetic classification of Poaceae includes a deep grade of three lineages: Anomochlooideae, Pharoideae, and Puelioideae, as well as the crown grasses represented by the BEP (Bambusoideae, Ehrhartoideae, Pooideae) [2] and PACMAD (Panicoideae, Aristidoideae, Chloridoideae, Micrairoideae, Arundinoideae, Danthonioideae) [3] clades. The PACMAD clade is of particular interest in this study because despite its paramount economic and ecological importance, phylogenetic relationships among its major lineages remain uncertain.

The sister group to the BEP clade has been variously defined as the PACC, PACCAD, PACCMAD, or PACMAD clade with different constituent subfamilies. A previous study [1] utilized the plastid gene sequence *ndhF*, which supported a monophyletic PACC (Panicoideae, Arundinoideae, Chloridoideae, Centothecoideae) clade, as well as indicating the polyphyletic nature of Arundinoideae. Subsequent work by the Grass Phylogeny Working Group (GPWG) [2] addressed weak support within and among the grass subfamilies by making use of informative characters in seven molecular datasets along with a morphological dataset. For comparative purposes we will refer to their results for three plastid sequences (*ndhF*, *rbcL*, *trnK/matK*), and not their eight-dataset analysis, as these did not differ in subfamilial arrangement, or provide further resolution.

The GPWG also increased taxon sampling over those of previous phylogenetic studies to include representatives of 62 genera, 30 of which fell within a group described under the newly established PACCAD (Panicoideae, Arundinoideae, Chloridoideae, Centothecoideae, Aristidoideae, Danthonioideae) clade [2]. Three taxa that nested within the PACCAD clade (*Eriachne*, *Gynerium*, and *Micraira*) were classified as *incertae sedis* (of uncertain placement). Arundinoideae were also found to lack unifying morphological or molecular synapomorphies to establish it as monophyletic. The genera classified as *incertae sedis* were analyzed further in a separate study with other representatives from *Eriachne* and *Micraira* through the use of 69 structural characters as well as *ndhF* and *rpl16* plastid sequences [6]. Their reinstatement of Micrairoideae as a distinct subfamily changed the PACCAD acronym to PACCMAD. With increased taxon sampling across Panicoideae and Centothecoideae in a subsequent study [7], it was concluded that Centothecoideae were paraphyletic with Panicoideae “…and the name should not have phylogenetic implications” (p. 1738). This study defined the constituent subfamilies of the PACMAD clade and established a backbone phylogenetic hypothesis against which deeper phylogenetic relationships could be explored.

The second GPWG constructed the most detailed grass phylogeny to date [4]. One of their major goals was to determine the number of C_4_ photosynthesis origins across the PACMAD clade. They analyzed 452 PACMAD species, encompassing two thirds of the genera within the clade using the same plastid markers from the previous GPWG study (*rbcL*, *ndhF*, *trnK*/*matK*). Multiple phylogenetic analyses and an increase in taxonomic sampling provided support for Aristidoideae as the sister subfamily to the rest of the clade. However, the relationship between Panicoideae and the CMAD (Chloridoideae, Micrairoideae, Arundinoideae, Danthonioideae) clade was only weakly supported (bootstrap (bs) value: 61 %, posterior probability (pp): 0.99), as well as the relationship between the MA (Micrairoideae, Arundinoideae) and DC (Danthonioideae, Chloridoideae) clades (bs value: 51 %, pp: 0.98). Furthermore, the arundinoids were only weakly supported as monophyletic.

Deep divergence time estimates of PACMAD grasses have been relatively few. This is partly because of the paucity of confidently dated grass fossils for use as calibration points at specific nodes [8, 9]. The fossils used for calibration include pollen, phytoliths, and spikelets [8, 10, 11]. Another contributing factor is the lack of a well-supported topology at the subfamilial level, especially for deep relationships within the PACMAD clade, which requires sufficient molecular sequences. Previous divergence estimates of the PACMAD clade are highly variable and have been examined in the stem Aristidoideae (28.8 to 61.1 mya), crown PACMAD (38 to 61.1 mya), and stem Panicoideae (26 to 42.1 mya) [8, 10–12]. These four studies used a relatively small number of molecular markers in their phylogenetic analyses and the lack of informative characters likely caused the topologies to vary.

Phylogenomic studies using complete plastomes from Poaceae have provided strong support for the relationships within and among other subfamilies [13–15]. This study addresses the weak support in previous research for deep nodes in the PACMAD clade by utilizing complete plastomes. A plastome from one arundinoid species (*Hakonechloa macra*) was sequenced using Sanger technology to provide a reference for annotation, and 12 additional complete plastomes were determined by next generation sequencing (NGS) methods for PACMAD taxa. Complete plastomes were analyzed phylogenomically and divergence dates estimated to seek potential selective causes for the PACMAD radiation. The analyses presented here utilize more phylogenetically informative characters and well supported phylogenomic relationships to provide a greater accuracy of divergence estimates through the use of complete plastomes.

Mitochondrial and plastome sequences may produce incongruent gene trees due to incomplete lineage sorting, recombination events, or potential elevated rates of substitution in grasses [16, 17]. Mitochondrial sequences were here explored with the goal of increasing maternally inherited character sampling among representative taxa. Mitochondrial sequence data were extracted and analyzed, as a source of potentially conserved characters, which have proven useful in determining subfamilial relationships in combination with plastome sequences [18–20].

## Results

### Outgroup selection, plastome

The PACMAD topology based on plastome data remained largely consistent across likelihood analyses conducted with different outgroups. In analyses of all but one outgroup taxon, Panicoideae were sister to the remaining PACMAD taxa. When the single taxon *Oryza sativa* was selected as the outgroup, Aristidoideae were sister to the remaining PACMAD taxa with a bs value of 56 %. Note that the use of *O. rufipogon* as an outgroup did not alter the topology in this way.

Outgroup selection greatly influenced support values for the position of Aristidoideae (Additional file 1: Fig. S1). Considering only single-taxon outgroups, the choice of *Puelia olyriformis* generated a bootstrap support (bs) value of 67 % for the PACMAD node. The use of the somewhat more closely related ehrhartoid species, *Oryza rufipogon*, increased the bs value to 76 %. When *Bambusa oldhamii* was used as the outgroup this node had a bs value of 80 %, but *Rhynchoryza subulata* provided the greatest support, a bs value of 99 %. *Bambusa oldhamii* was selected as the outgroup for the mitochondrial analysis since mitochondrial data were available for this species.

### Plastome characterization

The 13 new plastomes were largely conserved in gene content and organization. The short single copy (SSC) regions had ranges of 11,771 to 14,756 bp in length, long single copy regions (LSC) from 78,798 to 82,525 bp, and inverted repeat regions (IR) from 20,103 to 22,730 bp (Table 1). A unique deletion of ~1140 bp was found in the *rpl14* and *rpl16* region of *Hakonechloa macra*. This deletion eliminated all of *rpl16* as well as the first 70 bp of *rpl14* and the noncoding sequence between them. The deletion is found ~450 bp downstream of *rpl8* and ~100 bp upstream of *rps3*.

**Table 1:** Lengths of plastome subregions

Species	SSC	LSC	IR	%AT
*Aristida purpurea*	12540	80437	22723	62.5
*Centotheca lappacea*	12553	81451	22730	61.4
*Coleataenia prionitis*	12551	81886	22730	61.3
*Panicum virgatum*	12562	81659	22699	61.4
*Thysanolaena maxima*	12466	82173	22729	61.3
*Zea mays*	11771	82352	22748	61.5
*Elytrophorus spicatus*	12543	81221	21291	61.6
*Hakonechloa macra*	12743	80977	20103	61.3
*Monachather paradoxus*	12626	82525	21230	61.4
*Phragmites australis*	14756	82327	21268	61.3
*Eriachne stipacea*	12573	79849	21162	61.4
*Isachne distichophylla*	12650	81006	21063	61.5
*Micraira spiciformis*	12721	80073	21262	61.4
*Chaetobromus dregeanus*	12083	78798	20922	61.6
*Chionochloa macra*	12518	80888	21157	61.6
*Danthonia californica*	12346	79647	21099	61.6
*Neyraudia reynaudiana*	12695	80616	21028	61.6
*Rhynchoryza subulata*	12594	82029	20840	61.0

All lengths are reported in base pairs. The percent AT richness of each plastome is also reported. SSC: short single-copy; LSC: long single-copy; IR: inverted-repeat

### Plastome phylogenomic analyses

**Fig. 1:**
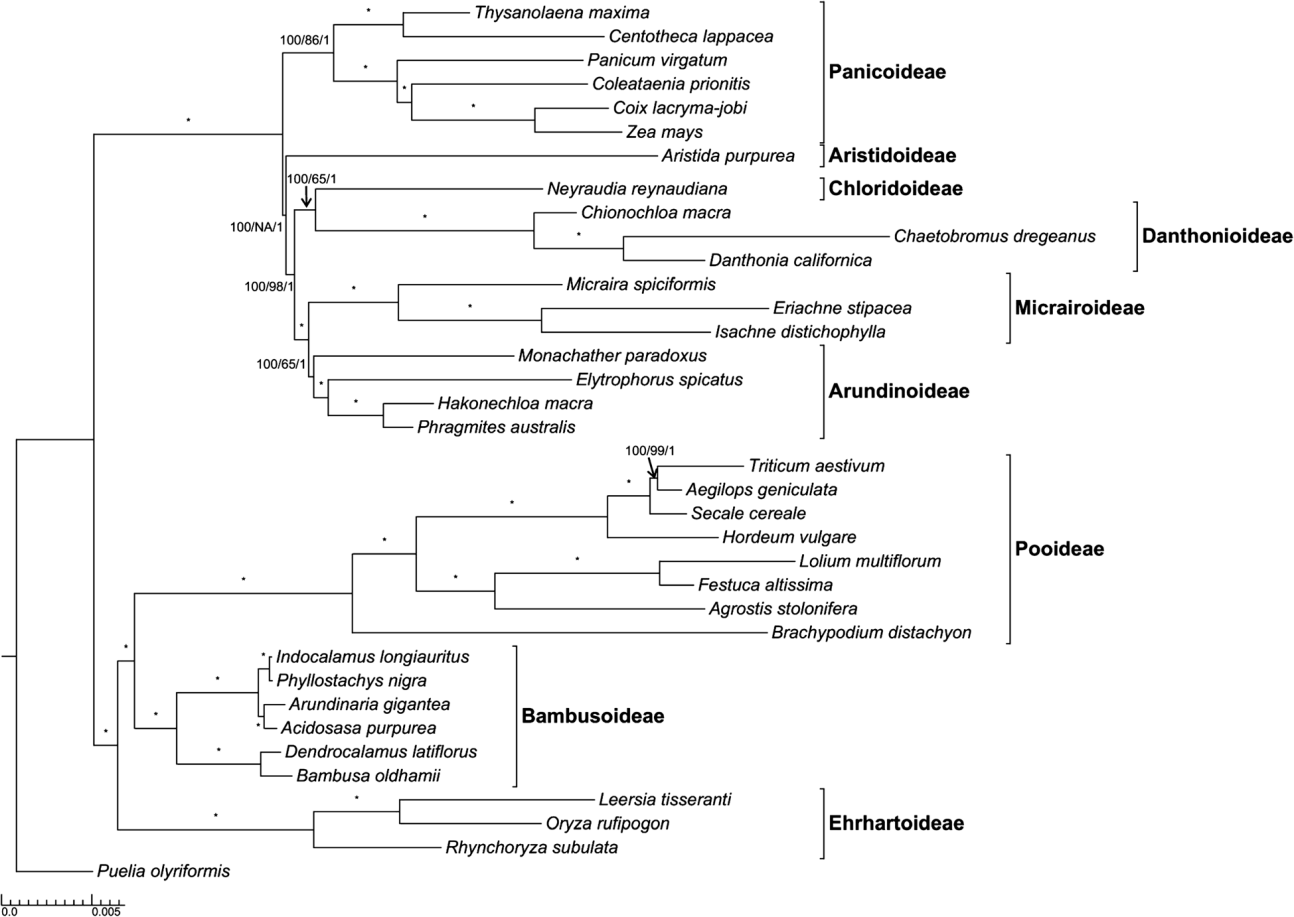
Maximum likelihood phylogram produced from a complete plastome analysis of 18 species of PACMAD grasses. Each node that was fully supported with a ML bs value = 100, MP bs value = 100 and a pp = 100 is labelled with *, except where noted (ML bs/MP bs value/pp). MP bootstrap values are marked NA in the one case where the topology differed

The maximum likelihood (ML) analysis produced a tree with − lnL = 274737.67. The tree had mean terminal branch lengths (0.009) more than 2.5 times greater than the mean of the internal branch lengths (0.0035). In the topology generated from the ML analysis, Panicoideae (six species) are resolved as the sister subfamily to the rest of the PACMAD clade (Fig. 1). The next subfamily to diverge is Aristidoideae, which is united with the CMAD clade with a bs value of 77 %. Chloridoideae is supported as sister to Danthonioideae with a bs value of 100 %. The sister relationship between Micrairoideae and Arundinoideae is also supported with a bs value of 100 %. *Elytrophorus spicatus* was embedded within the Arundinoideae and resolved as sister to the clade of *Hakonechloa macra* and *Phragmites australis* with maximum bs support. The chloridoid/danthonioid clade is supported with a bs value of 100 as sister to the arundinoid/micrairoid clade. Although the ML topology retrieved here was well-supported, the Shimodaira-Hasegawa (SH) test failed to reject the alternative hypothesis of Aristidoideae sister to the PCMAD clade (p < 0.151).The Bayesian inference (BI) topology was identical to the ML topology. The position of Aristidoideae and all other nodes in the topology were supported with posterior probability (pp) values of 1.0. The same data matrix under parsimony analysis had 10,779 parsimony informative sites. The maximum parsimony (MP) analysis produced a single tree of length 33,593 steps. The MP analysis had an ensemble consistency index of 0.5764 and retention index of 0.7911. The divergence order for the MP analysis varied slightly from the ML and BI analyses with Aristidoideae sister to the rest of the PACMAD subfamilies, followed by the Panicoideae. Bootstrap support values at each node were 100 with the exception of: the crown Arundinoideae (bs value = 65 %), crown DC (bs value = 99 %), crown CMAD (bs value = 98 %), and crown Panicoideae (bs value = 86 %). The sister relationship of two outgroup taxa, *Triticum aestivum* and *Aegilops geniculata*, was supported with a bs value of 99 % in the MP analysis. The partitioned analysis, using only 76 protein coding sequences, produced identical topological results with similar bootstrap support and will not be further considered here.

### Mitochondrial analysis

**Fig. 2:**
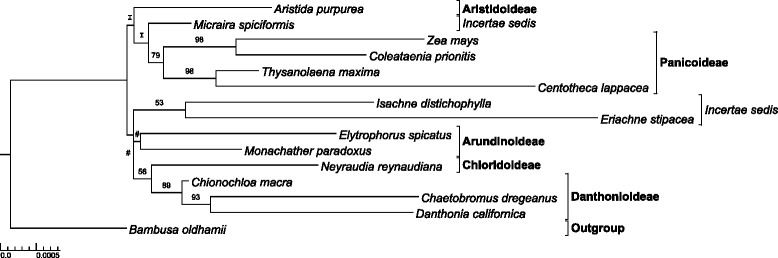
Maximum likelihood phylogram produced from analysis of assembled and aligned mitochondrial matR and seven intron sequences from 15 species. Branch lengths are proportional to the substitution rate along the branch. *Bambusa oldhamii* was selected as the outgroup. Bs values >50 and <100 are noted. Nodes labelled with # denote bs values <50. Each node marked “I” was incongruent with our ML plastome analysis topology (Fig. 1)

The mitochondrial analysis produced a tree with -lnL = 17041.15 (Fig. 2). Panicoideae were monophyletic, but with relatively weak support (bs value = 79 %). The sister relationships of *T. maxima* and *Centotheca lappacea* as well as *Z. mays* and *Coleataenia prionitis* were each supported with bs values of 98 %. A bs value of 89 % supported Danthonioideae as monophyletic. The sister relationship of Danthonioideae and Chloridoideae was retained, but with little support (bs value: 56 %). Arundinoideae, as sampled here, were characterized as monophyletic, but with bs value < 50 %.

The deep mitochondrial topology differed greatly from that of the plastome analysis (Fig. 2). Arundinoideae were sister to the Chloridoideae/Danthonioideae clade with a bs value < 50 % unlike the relationships in GPWG II [4] or this paper (Fig. 1). Micrairoideae were polyphyletic and all bs values placing these three species were lower than in the plastid ML tree. The deep nodes of the mitochondrial topology were substantially incongruent to those from the plastome analyses and were not used here in further analyses of the deep PACMAD nodes.

### Divergence date estimation

Divergence dates were estimated under two calibration scenarios (Table 2). The estimated divergence date at the crown PACMAD node was 32.44 [11.9, 50.6] mya and 32.74 [17.0, 45.2] mya for calibration sets one and two respectively, indicating that the use of the controversial phytolith in the outgroup had little impact on the estimated age of this node. The Aristidoideae divergence of 31.19 [12.8, 46.6] mya for the first set and 20.46 [10.6, 25.6] mya for the second set was the most variable between the two. In set two, the addition of another calibration point caused the stem and crown arundinoid divergences to decrease while the divergence dates of the crown micrairoid, danthonioid, panicoid and stem chloridoid lineages increased (Table 2). The crown panicoid divergence date of 20.24 [7.9, 36.8] mya for set one and 23.61[8.2, 36.8] mya for set two is also of note. Our estimated date for the crown Panicoideae is consistent with recent divergence estimates of Andropogoneae [21].

**Table 2:** Divergence estimations for two BEAST analyses

	Calibrations		Crown PACMAD	Arist	MADC	Arund/Micr	Chlor	Danth	Panic
1	(Chloridoideae: 14–19 mya)	Age	32.44	31.19	25.77	22.58	13.48	7.48	20.24
	(Crown Panicoideae: 7–8 mya)	95 % HPD Lower	11.89	12.75	10.33	9.80	7.63	1.80	7.93
		95 % HPD Upper	50.55	46.57	38.26	34.33	19.58	15.60	36.82
2	(Chloridoideae: 14–19 mya)	Age	32.74	20.46	19.08	17.31	14.43	12.99	23.61
	(Crown Panicoideae: 7–8 mya)	95 % HPD Lower	16.99	10.55	9.51	7.26	5.74	2.86	8.21
	(Crown BEP/PACMAD: 65–67 mya)	95 % HPD Upper	45.20	25.61	22.61	19.79	18.97	13.29	36.75

Respective fossil calibrations and estimated ages of each node are reported in mya. Arist: Aristidoideae; Arund/Micr: Arundinoideae + Micrairoideae; Chlor: Chloridoideae; Danth: Danthonioideae; Panic: Panicoideae

## Discussion

Short, deep branches of the PACMAD clade in our analyses are consistent with rapid radiations early in this group. Regardless of the cause, deep branches in the PACMAD grasses are difficult to resolve. The full plastome phylogenomic analyses of the PACMAD clade with hundreds of informative characters offer a clear advantage to understanding the deep divergences in the group. Full plastome analysis and estimation of divergence times allows for speculation on the cause of this accelerated diversification.

### Phylogenomic analysis

Phylogenetic analyses of rapid radiations in plant lineages tend to be challenging because long ingroup branches are connected only by short deep branches with relatively little phylogenetic information, which may hinder robust resolution of deep relationships [22]. When outgroups are on relatively long branches they can artifactually attract long ingroup branches and suggest erroneous relationships. The conflict between our ML and MP analyses is also suggestive of long-branch attraction to which parsimony is somewhat more susceptible [23].

With one exception, each ML analysis generated an identical ingroup topology. Long branch attraction tends to be indicative of homoplasious substitutions or possible elevated substitution rates, and may be responsible for the weakly supported result with *O. sativa.* This stands as a unique exception compared to all of the other outgroup combinations. Several outgroup taxa produced less support for the stem Aristidoideae. Outgroup taxa were selected for their relatively close relationships to the PACMAD grasses. *Bambusa oldhamii, Oryza rufipogon*, and *Rhynchoryza subulata* presented stronger support for this node than the more distantly related *Puelia olyriformis. B. oldhamii* exhibited a shorter terminal branch length than *R. subulata* and *O. rufipogon* [15], which was correlated with greater support at the stem Aristidoideae (80 %) that fell between those values when *R. subulata* (99 %) and *O. rufipogon* (76 %) were selected as outgroups. The inclusion of 18 outgroup taxa resulted in the addition of more phylogenetic information. Although support for the sister relationship of Panicoideae to the rest of the PACMAD clade was less than that of analyses with a single outgroup taxon (77 %), the larger outgroup allowed for greater confidence in the ingroup topology.

A significant past study on the systematics of Poaceae [4] provided weak support for the sister relationship of Panicoideae to the CMAD clade (bs value: 61 %) as well as between the micrairoid/arundinoid and danthonioid/chloridoid clades (bs values: 51 %). Although the taxon sampling of GPWG II included 452 PACMAD species, only three genetic markers of 600–800 base pairs (bp) each were analyzed. The phylogenomic methods here allow for an increase in the number of molecular markers by several orders of magnitude to provide additional informative sites and raise support values for the phylogeny.

In our most well-supported likelihood topology (Fig. 1), Panicoideae are sister to the other PACMAD grasses, and Aristidoideae are sister to the CMAD clade, but this relationship is not statistically different from the GPWG II topology by the SH test. The difficulty in discriminating between these two alternative topologies may be due to the rapid radiation of the PACMAD grasses. All subfamilies sampled with two or more species were recovered as monophyletic. Within Panicoideae, the two Andropogoneae (*Coix lacryma-jobi* and *Zea mays*) were sisters as were two species formerly characterized as centothecoids (*Thysanolaena maxima* and *Centotheca lappacea*) (bs values, both 100 %). Complete plastome sequence analyses were thus able to provide greater support for phylogenetic relationships and suggests that further sampling of complete plastomes from PACMAD taxa might be useful to address relationships at lower taxonomic levels. This analysis may be creating artifactual groups due to modest taxon sampling as compared to GPWG II [4], but greatly improves upon character sampling.

The ML topology of the mitochondrial data was substantially incongruent with our whole plastome phylogenies (Figs. 1, 2) and the relationships in the GPWGII analysis [4], especially at the deepest nodes. This may be due to the relatively low rate of substitution seen in plant mitochondrial genomes, or their tendency to recombine with fragments of other genomes/organisms that could link loci with different evolutionary histories [24]. Some subfamilies (Panicoideae, Danthonioideae) were retrieved with moderate support. Mitochondrial sequences may be more appropriately used as phylogenetic tools within subfamilies of PACMAD grasses.

### Divergence estimates

Previous studies have set out to determine divergence dates for PACMAD grasses using many taxa, but with a relatively small number of molecular markers. The increase in molecular data in this study allows for a more accurate assessment of divergence dates at deep nodes due to the presence of a greater number of phylogenetically informative sites. The age of the BEP/PACMAD clade was recently estimated to be 54.9 (±7.0) mya [9]. This estimate suggests that the BEP and PACMAD clades diverged at the approximate time of the Paleocene-Eocene Thermal Maximum (PETM) (55–56 mya) and the transition to the Eocene era. The Eocene is characterized as a period of cooling and drying, which led to forest fragmentation and created new or more extensive habitats for open habitat and forest margin species [25].

The divergence of the PACMAD subfamilies, which has been formerly estimated to fall between 38 mya [12] and 45 mya [8], was resolved at an age of 32.4 [11.9, 50.6] (95 % HPD lower and upper, respectively) mya in this analysis. The rapid radiation of the PACMAD clade according to this somewhat younger estimate occurs along the Eocene-Oligocene transition (EOT). Throughout the Eocene there was a global cooling trend following Antarctic glaciation events [26] as well as declining atmospheric CO_2_ [12]. These climatic changes influenced habitat diversification across the globe, increasing open habitats and forest margins for plant colonizations following the EOT.

Aristidoideae are almost exclusively open habitat grasses and the most parsimonious interpretation for the ancestral condition for the subfamily is that it was also an open habitat lineage [2]. If, as suggested by GPWG II, the Aristidoideae are sister to the other PACMAD grasses, then the exploitation of open habitats long preceded the radiation of the PACMAD clade for which there is no corresponding explanatory hypothesis [4]. In the context of the overall grass phylogeny, PACMAD habitat shifts are more parsimoniously interpreted if the sister group to other PACMAD species has the ancestral habitat type. Note that the deeply diverging lineages Puelioideae, Pharoideae, and Anomochlooideae, are exclusively found on forest floors [2]. Panicoideae comprise genera that occur in shady habitats, open habitats, and mixed habitats [2]. This kind of habitat diversity among species of the subfamily sister to the rest of the PACMAD clade would be expected if climatic changes promoted the radiation and diversification of open habitat species at the time of the EOT. Species composition of these descendants, which are resolved here as the sister group to the rest of the PACMAD lineage, would be expected to fill both shade tolerant and open habitat niches, which is seen in contemporary Panicoideae [4]. Notably, in our analysis the lineages sister to the rest of Panicoideae are forest margin (*Thysanolaena*) and shade tolerant (*Centotheca*) species [27], consistent with the hypothesis that the Panicoideae initially occupied forests and forest margins, and then radiated, possibly multiple times, into open habitats.

## Conclusions

Deep PACMAD relationships are here retrieved with greater support than in previous studies through the use of a phylogenomic approach. Our results support a PACMAD topology where Panicoideae is sister to the ACMAD clade allowing for further exploration of terminal relationships. It also offers a general phylogenomic approach for investigating rapidly radiating plant lineages.

Divergence estimates for the PACMAD clade provide insight into the putative role of climate changes leading to habitat diversification, which possibly triggered the rapid radiation of these grasses. The date of 32.4 mya for the initial radiation of the lineage is correlated with the EOT. Glaciation events and an overall global cooling trend throughout the Eocene led to environmental diversification via forest fragmentation and expansion of open habitats. These changes may have allowed grasses to rapidly speciate, hybridize, and ultimately dominate newly developed habitats.

## Methods

### Outgroup selection

Outgroup sampling with respect to taxon selection and number posed a challenge due to the potential sensitivity of the positions of Aristidoideae and Panicoideae as the sister group to the other PACMAD lineages. Initially, *Oryza rufipogon* (NC_022668) was selected as the outgroup, but support for Aristidoideae as sister to the CMAD clade was low (bs value 76 %). Another BEP representative, *Bambusa oldhamii* (NC_012927), was chosen as the outgroup and the topology remained consistent, but weak support was still seen at the stem Aristidoideae node (bs value 80 %). Up to 17 representative BEP taxa, as well as basal grade taxa (*Anomochloa marantoidea*, NC_014062; *Pharus latifolius* NC_021372; *Puelia olyriformis,* NC_023449), were combined in nine subsets to determine the effect of outgroup selection on ingroup topology and support*.* Ingroup topology proved to be stable across these analyses, although support for short deep branches was variable. Ultimately, the single outgroup species *Rhynchoryza subulata* (NC_016718; Ehrhartoideae) was found to produce results congruent to those of the largest outgroup species sets analyzed here.

### Taxon sampling

Taxa were sampled based on subfamilial membership to obtain representation for all major groups of interest. Taxa from Panicoideae include representatives of five major tribes, *Panicum virgatum* (Paniceae) (NC_015990), *Zea mays* (Andropogoneae) (NC_001666), and *Coleataenia prionitis* (Paspaleae). *Centotheca lappacea* (Centotheceae) and *Thysanolaena maxima* (Thysanolaeneae) were also included from the formerly recognized Centothecoideae [1, 2, 6], which [7] classified as Panicoideae. Three representative Arundinoideae, *Hakonechloa macra*, *Monachather paradoxus*, and *Phragmites australis,* which represent three major clades of Arundinoideae as retrieved by GPWG II were included [4]. One member of an arundinoid genus classified as *incertae sedis*, *Elytrophorus spicatus*, was also included in an attempt to resolve this arundinoid relationship [28]. Three taxa from the subfamily Micrairoideae were also analyzed to provide representation for the micrairoid/arundinoid clade. *Micraira spiciformis* (Micraireae), *Eriachne stipacea* (Eriachneae), and *Isachne distichophylla* (Isachneae) were chosen to represent three of the four tribes of micrairoids. Four genera within Danthonioideae were also included to represent the danthonioid lineage as well as a representative species of Aristidoideae, *Aristida purpurea* (Aristideae)*.* The only published plastome for a chloridoid species, *Neyraudia reynaudiana*, was also included in our analyses [29].

Mitochondrial sequence data for each taxon included in the plastome analysis that was sequenced using NGS methods were retrieved from the Illumina read files. Sequences from other taxa were retrieved from the NCBI database. Because of this, our mitochondrial sampling was limited to species that were sequenced using NGS and those represented in Genbank. For this portion of the study, the taxon set was limited to 15 species with *B. oldhamii* as the outgroup taxon.

### DNA extraction and sequencing

Leaf tissue samples were obtained for each species of interest (sources listed in Table 3) and dried with silica gel desiccant. Liquid nitrogen was used to lyse cells during manual homogenization of plant tissue to maximize DNA yields. Extractions were performed using the DNeasy Plant Mini Kit (Qiagen, Valencia, CA, USA) following the manufacturer’s protocol.

**Table 3:** Species newly sequenced for this study

Species	Subfamily	Voucher	GenBank Accession
*Aristida purpurea*	Aristidoideae	Saarela 605 (CAN)	KJ920224
*Centotheca lappacea*	Panicoideae	Sanchez-Ken s.n. (ISC)	KJ920225
*Coleataenia prionitis*	Panicoideae	Morrone 6195 (SI)	KJ920228
*Thysanolaena maxima*	Panicoideae	Clark & Gallaher 1717 (ISC)	KJ920236
*Elytrophorus spicatus*	Arundinoideae	Saarela 1685 (CAN)	KJ920230
*Monachather paradoxus*	Arundinoideae	Saarela 1629 (CAN)	KJ920235
*Eriachne stipacea*	Micrairoideae	Jacobs 8773 (NSW)	KJ920231
*Isachne distichophylla*	Micrairoideae	Morden 1227 (HAW)	KJ920233
*Micraira spiciformis*	Micrairoideae	Jacobs 8850 (NSW)	KJ920234
*Chaetobromus dregeanus*	Danthonioideae	Barker 978 (PRE)	KJ920226
*Chionochloa macra*	Danthonioideae	Buxton s.n. (CHR)	KJ920227
*Danthonia californica*	Danthonioideae	Saarela 860 (CAN)	KJ920229
*Hakonechloa macra*	Arundinoideae	Burke s.n. (DEK)	KJ920232

Subfamily, voucher information and Genbank accession are reported

The plastome of one species, *Hakonechloa macra*, was amplified and Sanger sequenced using grass-specific primers [30] and the general methods of [31]. Only one copy of the IR (inverted repeat) was sequenced as well as all four IR boundaries. The alternative methods of [32] were followed when amplifications failed, including the design and use of primers tailored to *H. macra* (Table 4). Amplifications were prepared for sequencing using the Wizard SV PCR Clean-up System (Promega, Madison, WI, USA). Sanger sequencing was performed at Macrogen, Seoul, South Korea. A complete plastome was manually assembled from the overlapping sequences and adjacent segments using Geneious Pro version 7.0.1 (Biomatters, Auckland, New Zealand). The other 12 plastomes not acquired through the NCBI nucleotide database were sequenced using NGS. Starting quantities of total genomic DNA from three DNA extracts (*Danthonia californica, Elytrophorus spicatus, Monachather paradoxus*) were determined with a Nanodrop 1000 (ThermoFisher Scientific, Wilmington, DE, USA) to be 1.5 μg each. DNAs were diluted to 2 ng/μl and sheared into 300 base pair (bp) fragments using a Bioruptor® sonicator (Diagenode, Denville, NJ, USA) in two 12 min periods, with inversion of tubes between them. DNA preparations were then purified and concentrated using the MinElute Extraction Kit (Qiagen Inc., Valencia, CA, USA). Single read libraries were prepared with the TruSeq low throughput protocol (gel method) following manufacturer instructions (Illumina, San Diego, CA, USA). Single-end sequencing was conducted on a HiSeq 2000 instrument (Illumina, San Diego, CA, USA) at Iowa State University, Ames, USA. Illumina reads were 99 bp in length.

**Table 4:** Species-specific Primers Designed for Hakonechloa macra

Primer Name	Sequence
1FHma	CATTACCCACTTGTCCGACTGTTGC
2RHma	CGATACTGGAACTCAGAGCATAGGAGG
12RHma	GCATGATATTGGGGAATCTCCTTGC
31FHma	GCTGCGTGTATAAGAGCCGAAATGGG
31RHma	GGCAACGAGCATCCAAAACCAAAAG
31FHma2	GCCCGAAATGGAGTGGGTCCTTCC
31RHma2	GGTTTTGGATGCTCGTTGCCGCTAG
39FHma	CCTTGGGGTAAAGAGTTTACACTGC
99RHma	CCTGTAGTAGGGATCTGGTTCACTGC
101FHma	GCGACCCCACAGGCTTGTACTTTCG
101RHma	CTTCGTCTACCCACTTCTCTTTCAGG
116FHma	GAACCTGGAGGAGTTAGGTATGTAGG
116RHma	GGAGTCGGCAAATGGACTGAGAATG
148FHma	GGCGTTTTTTTAGTGAGTCCTGG
148RHma	CTCTTTGAGAGACCCCGCTTATTGC
148FHma2	GAGACCGACCCACTTCCTATCTAG
169FHma	GCCGTTATTGTCGCAAGCATACGAC
169RHma	GCAGAATTTCTCTAGTAGGAGCGTC
169FHma2	CTCTATGGTTTGGGGCTTTAGCAGG
169RHma2	CACTCGGATAAACGCAGCAACACGG
184FHma	GCAACAGTCGGACAAGTGGGTAATG

An improved NGS method was used for the remaining nine species. Total genomic DNA extracts for the remaining taxa were diluted to 2.5 ng/μl in 20 μl water. The Nextera Illumina library preparation kit (Illumina, San Diego, CA, USA) was used to prepare libraries for sequencing and the DNA Clean and Concentrator kit (Zymo Research, Irvine, CA, USA) was used for library sample purification. Single-end sequencing was performed with the HiSeq 2000 instrument at the Iowa State University Sequencing Facility as above.

The reads were first quality filtered using DynamicTrim v2.1 from the SolexaQA software package [33] with default settings, and then sequences less than 25 bp in length (default setting) were removed with LengthSort v2.1 in the same package. The quality of the reads was then assessed using FastQC v0.10.1 (www.bioinformatics.babraham.ac.uk/projects/fastqc/). The complete quality trimmed set of reads was used for assembly.

### Plastome assembly and annotation

Plastome assembly was performed with entirely *de novo* methods. The Velvet software package [34] was run iteratively following previously established methods [26] to assemble reads into contiguous sequences (contigs). Contigs were scaffolded using the anchored conserved region extension (ACRE) method [29]. Conserved regions were identified using a grass family-wide alignment of plastomes. Any remaining gaps in the plastomes were resolved using contigs or reads by locating overlapping regions until the circular map was complete. Assembled plastomes were annotated in Geneious Pro by aligning them to a closely related and previously annotated reference plastome and transferring the annotations from the reference to the assembled plastome when the annotation shared a similarity of 70 % or above. Each coding sequence was then examined and necessary adjustments were made to preserve intron boundaries, reading frames, stop codons, or to identify pseudogenes. The IR boundaries were located using the methods of [13].

### Plastome analysis

Fully assembled plastomes were aligned in Geneious Pro using the MAFFT plugin [35]. Characters for which gaps were introduced in one or more sequences by the alignment were removed to reduce regions of ambiguous homology and one of the IR sequences was removed to reduce overrepresentation of duplicated sequence. This alignment was 96,493 bp in length after IR and gap removal. An ML analysis was performed using RAxML-HPC2 on XSEDE [36] and was accessed using the CIPRES science gateway [37]. The tree with the highest likelihood was obtained. Default parameters were used under a GTR model with estimation of the 25 per site gamma rate categories and all other free model parameters. A rapid bootstrap analysis was performed with 1,000 pseudoreplicates. The specified outgroup included 18 BEP taxa (Additional file 1: Fig. S1). The same methods were used for other combinations of outgroup taxa. A consensus bootstrap tree was produced with the Consense function of the Phylip software package [38] on CIPRES (Fig. 1). Tree files were visualized and edited using FigTree v1.4.0. [39]. Several other likelihood analyses were conducted using the separate outgroup taxa, *Puelia olyriformis*, *Bambusa oldhamii*, and *Oryza rufipogon*, but these analyses provided low bootstrap support (bs) values for the stem Aristidoideae. The SH test [40] was performed to determine if the ML topology obtained here differed significantly from the GPWG II hypothesis. The SH test was performed using PAUP* v4.0b10 [41] to determine if the topology with Aristidoideae as the sister group to the other PACMAD subfamilies [4] could be rejected by the data analyzed here.

A BI analysis was performed using MrBayes 3.2.2 on XSEDE [42], which was accessed using the CIPRES science gateway. The Markov chain Monte Carlo (MCMC) analysis was run twice at 10,000,000 generations each. This was run twice with a burn-in of 5,000,000 generations. The analysis was run until completion, and the average standard deviation of split frequencies was < 0.00001.

Branch and bound maximum parsimony (MP) analyses were performed using PAUP* v4.0b10 [41]. An MP bootstrap analysis with 1000 pseudoreplicates, each with 10 random addition sequence replicates, was also performed.

A partitioned analysis was performed by concatenating 76 protein coding sequences from each full plastome. The partitioned matrix was analyzed using identical methods as the full plastome analysis.

### Mitochondrial analysis

Mitochondrial sequences were assembled from the same NGS read files and analyzed in a likelihood framework using the same utilities as the plastome analysis. The *matR* gene sequence was chosen based on prior use in phylogenetic analyses [18]. Six relatively variable intron sequences [19] were also chosen: *nad1* intron 2, *nad4* intron 1, and *nad7* introns 1, 2, 3, and 4. Each intron sequence had less than 99 % pairwise identity when aligned (Table 5). Mitochondrial sequences from *Zea mays* and *Bambusa oldhamii* were obtained directly from GenBank. A reference mapping analysis was performed for each species that was included in the NGS libraries by mapping each read file to each coding sequence of interest from the full mitochondrial sequence of *Zea mays*. The consensus sequences from each reference mapping were examined for missing nucleotide sites and for frameshift mutations, where applicable, to detect erroneous assemblies. The corresponding *matR* and intron sequence data (Table 5) were assembled, concatenated, and aligned for each species. ML analyses were performed on these data as before, but with *B. oldhamii* as the outgroup, to generate a ML tree (Fig. 2).

**Table 5:** Mitochondrial sequence analysis

Locus	Percent nucleotide identity
*mat-r*	99.3
*cox2* intron	91.1
*nad1* intron 2	97.4
*nad4* intron 1	98.1
*nad7* intron 1	95.5
*nad7* intron 2	98.3
*nad7* intron 3	98.9
*nad7* intron 4	98.0

Sequence identity between mitochondrial loci across a subset of the taxa in this study. The subset includes *Bambusa oldhamii* (NC_012927)*, Zea mays* (NC_001666) and all species in which plastomes were generated in this study using NGS

### Divergence estimates

Divergence dates were estimated using BEAST v2.1.2 [43] and parameters were set in BEAUTi. Preliminary divergence dates with different combinations of calibrations and different prior distributions were estimated. There are few fossils useful for calibrations of PACMAD grasses and dates are sometimes contradictory. Two divergence analyses were selected and presented here to show the maximum range of estimated dates. Both estimates were each given two different seed values and chain lengths of 10 million for a total of 20 million generations each. Priors were set to constrain relationships that preserved the topology generated by the ML plastome analysis with *Rhynchoryza subulata* as the outgroup. The substitution model utilized was GTR + G + I under an uncorrelated relaxed log-normal clock. For each of these a log-normal distribution was selected. Parametersolver v3.0 [44] was used to calculate the mean and variance of these log-normal prior distributions. Fossil estimates for calibration of each run differed to evaluate the effect of a potentially controversial fossil in the outgroup. A total of six fossils were used to calibrate the analyses. The first run was assessed using a lower bound at the stem Chloridoideae node of 14 mya calibrated with a fossil identified as a member of Chloridoideae, due to the shape of the stomatal subsidiary cells and silica bodies [45]. There has been some debate, however, on the taxonomic identity of this fossil to the genus *Distichlis* upon further microanatomical evaluation [46]. However, our use of this fossil here is as a calibration point at the crown Chloridoideae node. An upper bound of 19 mya was placed at the stem Chloridoideae node using a chloridoid phytolith fossil [47]. The core Panicoideae (Panicodae) [28] were also constrained for the first run with a lower bound of seven mya using a *Setaria* fossil, and upper bound of eight mya with a fossil assigned to *Dichanthelium* sp. [48]. The second BEAST run was conducted using the same calibrations as the first run, but with the addition of a lower bound on the crown BEP/PACMAD node of 65 mya and an upper bound of 67 mya using Oryzeae phytolith fossils [10]. The use of phytoliths as calibrations for estimation of divergence times in Poaceae is somewhat controversial [49]. Comparing two sets of analyses, with and without the phytoliths allows evaluation of the impact of this calibration on the divergence estimation. This calibration strategy incorporated taxonomically identified fossils for PACMAD grasses that are available. It also evaluated the effect of using the Oryzeae phytolith and associated cuticle fossils for the outgroup, which have been shown to produce date estimates that are incompatible with those from other data [9]. The tree and log files produced from the BEAST analysis were combined with Logcombiner v2.1.2 and convergence assessed using Tracer v1.6 [50]. FigTree v1.4.2 [39] was used to view and edit the combined tree file generated for each run of BEAST with a burn-in of 20 percent. Divergence estimates for nodes relevant to the deep relationships of PACMAD grasses are given (Table 2).

### Availability of supporting data

The data set(s) supporting the results of this article are available in the TreeBase repository, http://purl.org/phylo/treebase/phylows/study/TB2:S17812. All nucleotide sequences were deposited in the NCBI GenBank repository. Accession numbers can be found in Table 3.

## Additional file

Additional file 1:
**Schematic outlining the effects of outgroup selection on subfamilial relationships within the PACMAD clade.** The included outgroups for each estimation is shown along with the subfamily that is sister to the rest of the PACMAD clade. Additionally, the ML bootstrap support for this sister relationship is included. Figure S1. Outgroup taxon subsets chosen for phylogenomic analyses including total number and identities of outgroup species. Bootstrap values uniting all taxa sister to the deepest diverging clade, which is either Panicoideae or Aristidoideae, are shown. Generalized tree of phylogenetic relationships among Poaceae is indicated above.

## Abbreviations

ACRE: anchored conserved region extension
BEAST: Bayesian evolutionary analysis sampling trees
BEAUTi: Bayesian evolutionary analysis utility
BEP: Bambusoideae Ehrhartoideae Pooideae
CIPRES: cyber infrastructure for phylogenetic research
CMAD: Chloridoideae Micrairoideae Arundinoideae Danthonioideae
DC: Danthonioideae Chloridoideae
EOT: Eocene-Oligocene transition
GPWG (II): grass phylogeny working group (II)
GTR + G + I: general time reversible plus gamma distribution plus proportion of invariant sites
IR: inverted repeat
LSC: long single copy
MA: Micrairoideae Arundinoideae
MAFFT: multiple alignment using fast fourier transform
MCMC: Markov chain Monte Carlo
ML: maximum likelihood
MP: maximum parsimony
NGS: next generation sequencing
PAUP*: Phylogenetic Analysis Using Parsimony * and other methods
PACC: Panicoideae Arundinoideae Chloridoideae Centothecoideae
PACCAD: Panicoideae Arundinoideae Chloridoideae Centothecoideae Arundinoideae Danthonioideae
PACCMAD: Panicoideae Arundinoideae Chloridoideae Centothecoideae Micrairoideae Arundinoideae Danthonioideae
PACMAD: Panicoideae Arundinoideae Chloridoideae Micrairoideae Arundinoideae Danthonioideae
PETM: Paleocene-Eocene thermal maximum
SSC: short single copy
