# Supplementary figures and images for: Resolving deep relationships of PACMAD grasses: a phylogenomic approach
Source: BMC Plant Biol. 2015 Jul 11;15:178. doi: 10.1186/s12870-015-0563-9 (PMC4498559; doi:10.1186/s12870-015-0563-9)

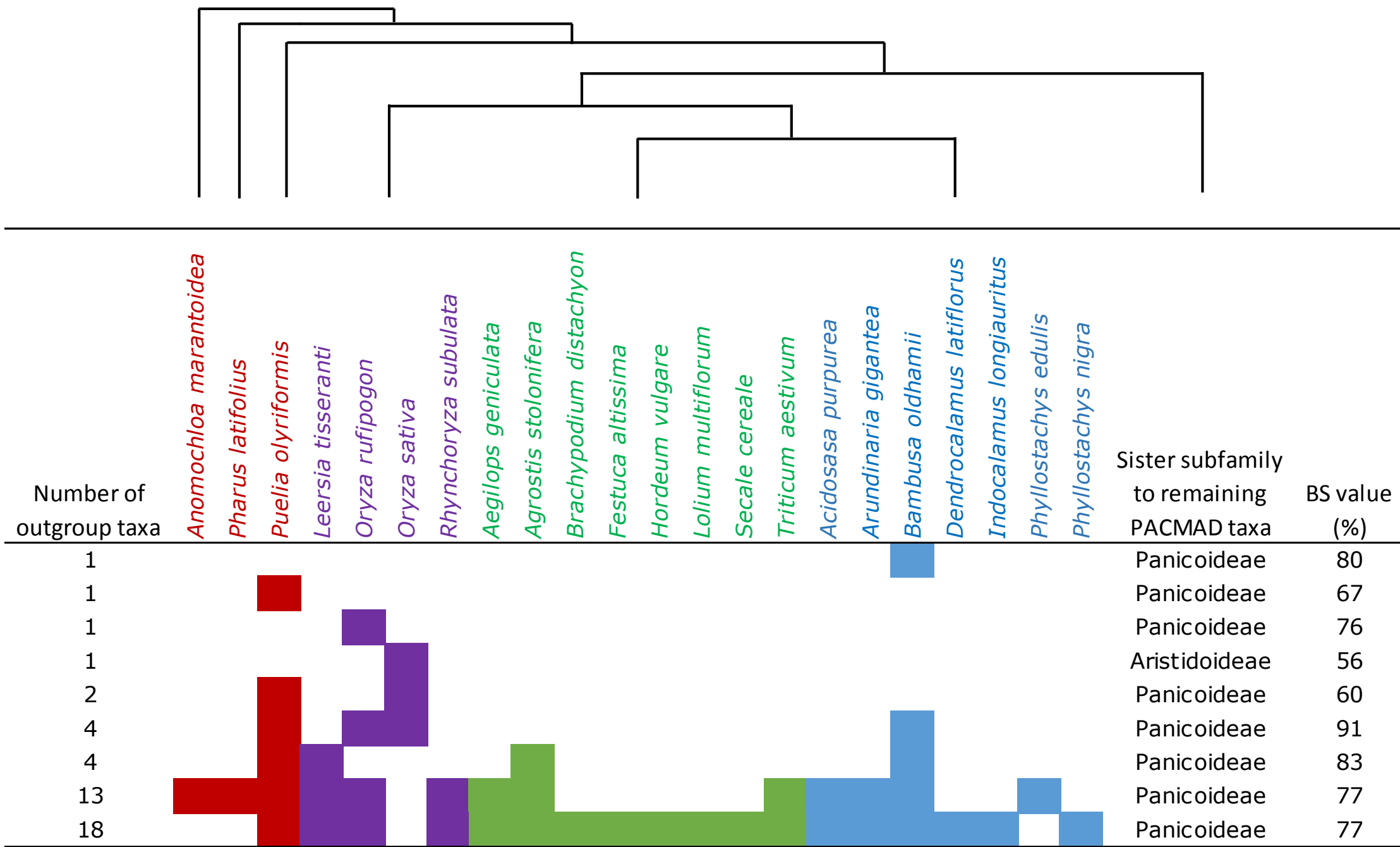

Supplement: Additional file 1: — Schematic outlining the effects of outgroup selection on subfamilial relationships within the PACMAD clade. The included outgroups for each estimation is shown along with the subfamily that is sister to the rest of the PACMAD clade. Additionally, the ML bootstrap support for this sister relationship is included. Figure S1. Outgroup taxon subsets chosen for phylogenomic analyses including total number and identities of outgroup species. Bootstrap values uniting all taxa sister to the deepest diverging clade, which is either Panicoideae or Aristidoideae, are shown. Generalized tree of phylogenetic relationships among Poaceae is indicated above. [file 12870_2015_563_MOESM1_ESM.pdf]
